# Membrane Based Measurement Technology for *in situ* Monitoring of Gases in Soil

**DOI:** 10.3390/s90200756

**Published:** 2009-02-02

**Authors:** Detlef Lazik, Sebastian Ebert, Martin Leuthold, Jens Hagenau, Helmut Geistlinger

**Affiliations:** UFZ - Helmholtz-Centre for Environmental Research, T.-Lieser-Strasse 4, 06120 Halle, Germany; E-Mails: martin.leuthold@ufz.de; jens.hagenau@ufz.de; sebastian.ebert@ufz.de; helmut.geistlinger@ufz.de

**Keywords:** Green house gases, CO_2_-sequestration, Gas, Monitoring, Membrane, Perm-selectivity

## Abstract

The representative measurement of gas concentration and fluxes in heterogeneous soils is one of the current challenges when analyzing the interactions of biogeochemical processes in soils and global change. Furthermore, recent research projects on CO_2_-sequestration have an urgent need of CO_2_-monitoring networks. Therefore, a measurement method based on selective permeation of gases through tubular membranes has been developed. Combining the specific permeation rates of gas components for a membrane and Dalton's principle, the gas concentration (or partial pressure) can be determined by the measurement of physical quantities (pressure or volume) only. Due to the comparatively small permeation constants of membranes, the influence of the sensor on its surrounding area can be neglected. The design of the sensor membranes can be adapted to the spatial scale from the bench scale to the field scale. The sensitive area for the measurement can be optimized to obtain representative results. Furthermore, a continuous time-averaged measurement is possible where the time for averaging is simply controlled by the wall-thickness of the membrane used. The measuring method is demonstrated for continuous monitoring of O_2_ and CO_2_ inside of a sand filled Lysimeter. Using three sensor planes inside the sand pack, which were installed normal to the gas flow direction and a reference measurement system, we demonstrate the accuracy of the gas-detection for different flux-based boundary conditions.

## 1. Introduction

There is an increasing demand for cost-effective and long-term stable measuring systems for gas monitoring in the environment [1, 2]. Beside traditional monitoring tasks (e.g., in research, emission analysis and safety) carbon capture and storage (CCS) develops to an important new application field for subsurface gas monitoring [3-6].

The analysis of CO_2_ gas comprises a long history going back 180 years with the development of several chemical and physical methods such as: gas chromatography, infrared analysis, ^14^C isotope measurement, mass spectrometry, FT-IR spectroscopy, gas diffusion-flow injection (GD-FIA) or continious flow systems based on photometric detection with various pH indicator systems, conductimetric sensors, thermistors and acoustic detectors [7].

Lewicki and Oldenburg [8] show by numerical investigations that monitoring of CO_2_ in the subsurface has greater potential to detect and quantify gas dynamics in heterogeneous ground than above-ground techniques. But up to now the development of a suitable measurement system for *in situ* gas monitoring remains to be a challenge, for both scientific and technical reasons. With respect to the heterogeneity of natural systems membrane based monitoring techniques particularly those based on polymers, gain increasing importance for environmental gas measurement.

Typically, membranes are used as a gas-permeable phase boundary. Based on this approach a gas saturometer was introduced already in 1975 to measure the equilibrium gas pressure for a given dissolved gas in a liquid. This technique is still available as *Total Dissolved Gas* sensor [9]. Numerous different applications combining standard analytical techniques and phase separation were developed e.g., [10-13]. Due to its low interaction such combinations of standard analytics with phase separating tubes have a significant importance for the *in situ* measurements.

The measurement behind a phase separating membrane requires the equilibrium for all permeating substances and therefore, a high permeability of the membrane would be preferable. On the other hand, low gas permeability is required to conserve the equilibrated gas constitution inside the membrane tube during its transport to the analytical device. This problem of optimization restricts the temporal resolution of the readings and the spatial extend of such a measurement system.

To overcome this limitation, we developed a flux-based measurement method [14] operating near the dynamic equilibrium, which is reached fast in contrast to thermodynamic equilibrium. The gas selectivity of membranes is used as sensory principle and no transport of some gas sample towards an analytical device is required. The robust method is applicable for quantification of the constitution of a multi-component gas [15] e.g., in soils, aquifers or bodies of water.

One objective of this paper is to demonstrate theoretically the equivalence of a continuous (volume-based) application of the sensor with the discontinuous (pressure based) method. In many practical cases one is interested in the concentration of only one gas component within a given gaseous or liquid phase. Therefore, we present a new concept for a single component analysis, which is a special case of the multi-component theory and which is the main objective of our paper. For this special case the constructive effort can be reduced and the sensor handling becomes relatively simple. We demonstrate the application of the single component analysis for monitoring of O_2_ and CO_2_ in a water-unsaturated soil.

## 2. Theory

### 2.1. Basic Concept of Permeation Based Gas Detection

According to the solution-diffusion model the permeation of a single gas through a dense tubular membrane proceeds in several steps. In the first step, gas is adsorbed from adjacent space at the outer membrane surface at *R_a_* [m] (outer membrane radius). Once the gas molecule is adsorbed, desorption or absorption will occur depending on the energetics of the surface. Absorption (which is considered as dissolution process) is the rate limiting step compared to the fast adsorption process. Inside the membrane the gas molecules diffuse according to the concentration gradient along the membrane radius. Its flux density *j* (*r,t*) [mol/m²/s] is described by Fick's first law *j*(*r,t*) = −*D* · ∇ *C* (*D* [m²/s] – diffusion coefficient of gas, r [m] – radius, t [s] – time). If the gas molecules reach the inner membrane surface at *R_i_* [m] (inner membrane radius), the mass transfer proceeds in reverse order: gas leaves the membrane phase and is subsequently desorbed into the gas phase.

Since both the adsorption-desorption processes and the gas diffusion processes outside the membrane are fast compared to the diffusion process within the solid membrane phase, an adsorption-desorption equilibrium and constant concentrations in both adjacent gas spaces can be assumed.

At sufficiently low concentrations, the generally non-linear adsorption isotherm can be approximated by a linear Henry isotherm *C*|*_R_a__* = *S^a^ p^a^ χ^a^ /(RT)* where *C*|*_R_a__* [mol/L] is surface concentration at the membrane, *p^a^* [Pa] denotes gas pressure in the external space (index ‘a’), *x^a^* [mol/mol] is the unknown mol fraction of gas and *S^a^* [m³(gas)/m³(membrane)] is solubility of the gas in the membrane which is related to the dimensionless inverse Henry constant (R = 8.3145 J/K/mol – gas constant, T [K] – temperature). The corresponding boundary condition for the interior space (index ‘i’) is given by *C*|*_R_i__* = *S^i^ p^i^ χ^i^ /(RT)* Furthermore, we only consider symmetrical membranes so that: *S* = *S^i^* = *S^a^*. For constant boundary conditions a dynamic equilibrium will be establish. Near this steady state the gas flow 2*π·r·L·j* (*r*) [mol/s] through the membrane will be constant (*L* [m] – length of the tubular membrane). Assuming that both the solubility and the diffusion coefficient are independent of the concentration, the number of moles *dv* that permanently permeate the membrane in the time *dt* is:

(1) $$d\nu =\frac{Pp^{a}}{RT}\frac{2\pi \cdot L}{\ln(R_{a}/R_{i})}(\chi^{a}-\gamma \chi^{i})\cdot dt,$$

where the material parameter *P* = *SD* [m²/s] is called permeability and *γ* = *p^i^/p^a^* is the ratio of gas pressures inside and outside the membrane tube.

Using the ideal gas law *V*_0_ (*p*_0_ + *dp*) = *p*_0_ (*V*_0_ + *dV*) = *RT* (*v*_0_ + *dv*) where V_0_ [m³] is the volume, *p*_0_ [Pa] is the pressure, and *v*_0_ [mol] the number of moles inside the measuring membrane tube (index ‘0’ indicates the initial state) one can substitute *v*_0_ in Equation (1) to obtains two measurable quantities: the volume change for isobaric conditions (*dV* = *RT/p*_0_·*dv*) and the equivalent pressure change (*dp* = *RT/V*_0_·*dv*) for isochoric conditions.

### 2.2. Multi Gas Analysis

Different measurement methods can be used to determine this change of pressure or volume. In principle, a discontinuous or a continuous procedure can be implemented. In both cases the dynamic equilibrium will be established by purging interior of the tubular membrane by a gas of known composition.

#### Discontinuous (isochoric) method

At time *t* = 0 (start of pressure measurement) the tubular membrane will be closed at its ends by valves.

We assume that superposition holds for the permeation of the different gas components (index ‘*k*’) of a multi-component system (e.g., soil air). Applying Dalton's law the resulting isochoric pressure change inside the tube is

(2) $$\frac{dp}{dt}=\sum_{k=1}^{n}\frac{dp_{k}}{dt}=g\;P_{s}\;p^{a}\sum_{k=1}^{n}f_{ks}\;(\chi_{k}^{a}-\gamma \chi_{k}^{i}),$$

where *f_ks_* = *P_k_/P_s_* is the perm-selectivity coefficient (defined with regard to a component *k* = *s* of the purging gas) and the geometrical properties of the tubular sensor are combined to the geometry factor *g* [1/m²]

(3) $$g=\frac{1}{V_{0}}\frac{2\pi \cdot L}{\ln(R_{a}/R_{i})}.$$

Recording the time-dependent pressure curve for *t* > 0 and approximating the discrete readings by a polynomial *F_p_* = Σ*_p_ a_p_* · *t^p^* the pressure change is determined by the limiting value

(4) $$a_{1}=\frac{dp}{dt}={\frac{dF_{p}}{dt}|}_{t\to 0},$$

where the dynamic equilibrium was still valid.

#### Continuous (isobaric) method

Steady state is continuously conserved by purging the tubular membrane. In analogy to Equation (2) the volume change near the dynamic equilibrium is

(5) $$\frac{dV}{dt}=\sum_{k=1}^{n}\frac{dV_{k}}{dt}=\frac{V_{0}}{p_{0}}\frac{dp}{dt}.$$

The diffusive gas flow through the membrane can be measured in terms of the change of the purging gas flow *dV/dt* = *Q_out_* – *Q_in_* [m³/s] between the inlet (*Q_in_*) and the outlet (*Q_out_*) of the tubular membrane. Using Equations (4) and (5) one again obtains:

(6) $$a_{1}=\frac{p_{0}}{V_{0}}(Q_{\text{out}}-Q_{\text{in}}).$$

Our measurement relay on either: the pressure changes with respect to the ambient pressure conditions or the volume change with respect to the purging gas flow. Both signals could be small with respect to external perturbations. Therefore, to improve the accuracy, the measurements could be related to a reference system. As the simplest case this is a non-permeable tube (e.g., a stainless steel capillary) having the same geometrical properties and situated in the same environment as the permeable membrane.

Combining Equations (4) and (2) a general equation for the gas analysis can be derived for both the methods:

(7) $$\sum_{k=1}^{n}\;f_{ks}\;p^{a}(\chi_{k}^{a}-\gamma \chi_{k}^{i})=\frac{a_{1}}{g\cdot P_{s}}.$$

Using *n* membranes of different perm-selectivities $f_{ks}^{j}$, a system of linear algebraic equations is obtained allowing for the determination of the unknown partial pressures ${\text{p}}_{k}^{a}=p^{a}\chi_{k}^{a}$.

### 2.3. Single Gas Analysis

The rank of this equation system can be reduced by using a-priori information (i.e. boundary conditions). For example, a known gas phase pressure *p^a^* can be used to substitute the partial pressure of a single gas component [15]. Additional relations between different gas components will further reduce the rank of the matrix.

The adsorption-desorption equilibrium of any gas component at the membrane surfaces supports the following statistical argument: The (ideal) gas molecules compete with each other for sorption sites on the membrane surface. Therefore, the change of a single component at $\lambda =\Delta \chi_{x}^{a}$ causes a shift of the mean surface concentration of all components depending on their total concentration. Inside of an open system (e.g., unsaturated soil) the gas pressure should be independent of local changes of partial pressures. Thus, for a known composition of the local gas phase $\sum \chi_{i}^{a}(t_{0})$ (e.g., background composition of air for *t*_0_) one obtains $\lambda (t)+\left(1-\lambda (t)\right)\sum \chi_{i}^{a}(t_{0})=\sum \chi_{i}^{a}(t)=1$, and by combination with Equation (7) the outer change of mol fraction is:

(8) $$\Delta \chi_{x}^{a}(t)=\frac{\frac{a_{1}(t)}{p^{a}\;g\;P_{x}}-\sum f_{kx}\;(\chi_{k}^{a}(t_{0})-\gamma \;\chi_{k}^{i}\;(t_{0}))}{1-\sum f_{jx}\;\chi_{j}^{a}(t_{0})}.$$

In Equation (8) the perm-selectivities are rearranged with respect to the permeability of the considered gas (index ‘x’). Thus, one finds the partial pressure by:

(9) $${\text{p}}_{x}^{a}(t)={\text{p}}_{x}^{a}(t_{0})+\Delta \chi_{x}^{a}(t)\left(p^{a}-{\text{p}}_{x}^{a}(t_{0})\right).$$

Equations (8) and (9) show linearity of type *p_x_* = *k_1_a_1_* + *k_2_* where *k_1_* and *k_2_* are constants that have to be calibrated. Note that Equation (9) is still valid in case the background composition does not contain gas x.

## 3. Lysimeter Experiment

**Figure 1.**
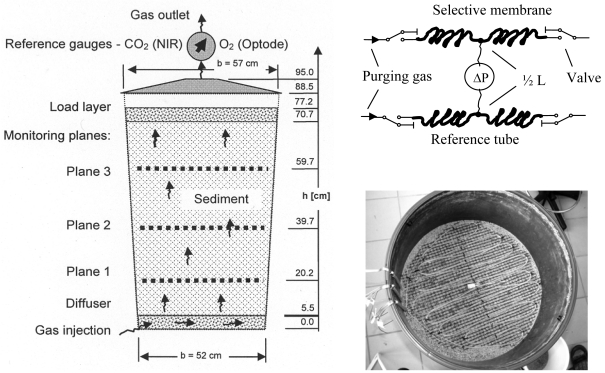
Design of the lysimeter for gas monitoring. Left: sketch of the vertical cross section of the lysimeter. Right: sketch of the selective membrane and the reference tube (top) and top view of the first monitoring plane (bottom) where the tubes are attached to a gauze (1 × 1 cm mashes) to fix their position.

A lysimeter experiment was designed to investigate the accuracy of the sensor under controlled conditions in the lab prior to *in situ* measurements in soil. The lysimeter (Figure 1) was filled by 238 kg dry medium sand (0.1 - 1 mm particle size). During filling three equidistant monitoring planes were installed horizontally. A diffuser (porous PE-sheet over a 21 kg gravel layer) at the bottom of the lysimeter guaranties a homogeneous gas flow. At the top a 28 kg gravel layer was inserted to stabilize the sand pack. Both sediments were divided by gauze (2 mm mashes) to prevent mixing. The lysimeter was closed and sealed.

The gas inlet at the bottom was connected to a set of calibrated mass flow controllers (MFC 8712, Bürkert Fluid Control Systems), which were used to define the composition of the continuously injected gas phase. The outlet of gas was in the centre of the top cover of the lysimeter. Reference gauzes for O_2_ (fiber-optic oxygen meter, Fibox 2, www.PreSens.de) and CO_2_ (near infrared, BCP-CO2, www.getsens.com) were installed near the outlet.

Each horizontal monitoring plane consists of a 6 - 7 m meander-like membrane tube. We used a commercial polydimethylsiloxane tubing (*R_i_* = 0.75 mm, *R_a_* = 1.75 mm, perm-selectivity's: *f*_O2/N2_ = 1.97, *f*_CO2/N2_ = 9.89 [16]) as sensor membrane. The individual membrane tubes were connected with valves (positioned outside the lysimeter) by stainless steel capillaries (1 mm aperture). The pressure difference between the sensor and the reference tube was measured outside the lysimeter by a pressure sensor (PCLA12X5D1, operating pressure 0 … ±12.5 mbar, www.sensortechnics.com) which was connected using stainless steel capillaries (1 mm aperture). The valves allowed to close the membrane tubes and to purge it by dry air. To quantify *a*_1_ the pressure development inside the closed tubes was recorded by the pressure sensor over 20 s.

In principle, the membrane sensors could be calibrated for single-gas analysis according Equation (9) using the extensive data sets available in literature. It should be noted that the actual material properties may differ from those in literature, due to the individual technological process of tube manufacturing and the actual chemical membrane formulation. Therefore, to enable the usage of ordinary, commercial available tubing, the membrane sensors were adjusted for the gas component of interest by simple 2-point calibration of *p_x_* = *k_1_a_1_* + *k_2_* prior to the experiments using the MFCs.

Flow rate and composition of the input gas was controlled by a PC, which was also used for the on-line conversion of pressure readings into partial pressures. Different gas mixtures were injected at the bottom of lysimeter with a constant flow rate of 0.5 L/min. They were analyzed within the monitoring planes using the calibrated membrane sensors. The time resolution of the measurement was set to about 6 min.

## 4. Results and Discussion

The first two experiments show the detection of O_2_ and CO_2_ within the monitoring planes for step-like input functions of the individual gas. To demonstrate the wide area of potential applications the concentration ranges were chosen with respect to typical scales of environmental applications.

**Figure 2.**
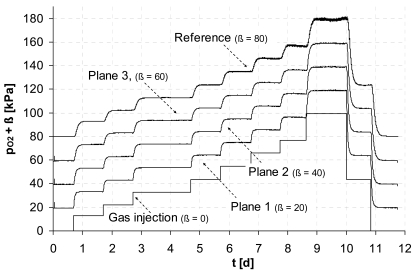

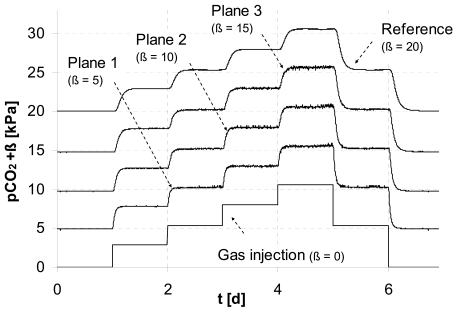
Detection of *p*_*O*2_ mixed with N_2_ (top) and *p*_*CO*2_ mixed with air (bottom) by the individual monitoring planes for a stepwise gas injection. For better readability the records of the different monitoring planes were separated by ß.

A wide concentration range of dissolved oxygen is of interest e.g., to control biosparging, a common technology applied for remediation of contaminated groundwater, which uses the injection of air to enhance the activity of microbes. In Figure 2 (top) a perfect match at the relevant concentration range for O_2_ mixed with N_2_ (to simulate groundwater-near gas composition) is illustrated between the new measurement technique and the reference optode within all monitoring planes.

An excellent agreement was also found for monitoring pCO_2_ (Figure 2, bottom). In this example we simulate a typical concentration range of CO_2_ mixed with air, which can be expected from aerobe microbial activity in soils.

Using the steady states (plateaus) where the gas concentration is the same throughout the entire experimental system, we estimate a mean statistical error for that first *in situ* sensor test of less then 2% with respect to the reading. The standard error (exemplarily estimated for the data from plane 1) of the regression against the individual reference sensors was for the O_2_-measurement less than 0.7 kPa (range 0 - 100 kPa) and for the CO_2_-measurement less than 0.08 kPa (range: 0 - 10 kPa). This smaller error of CO_2_-measurement can be mainly attributed to the higher selectivity of CO_2_ (*f*_CO2/N2_ = 9.89) with respect to the ones of O_2_ (*f*_O2/N2_ = 1.97) of the used membrane material.

**Figure 3.**
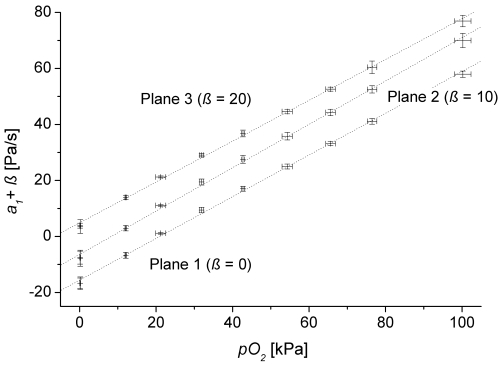
Correlation of pressure change *a*_1_ at the individual monitoring planes (see Figure 1) with the partial pressure *P*_*O*2_ measured by the reference optode. For better readability the error bars were formed by the 3-fold standard deviation for both *a*_1_ and *p*_*O*2_. The records of the different monitoring planes were separated by ß.

As a prerequisite of the applied theory the gas permeation through the membrane need to be independent of the concentrations within the observed measurement range so, that linearity between concentration and pressure change can be assumed. However it is widely known from literature that this independence is only given for small concentrations. Hence, the corresponding limits need to be known. To investigate this critical aspect the measured coefficients *a*_1_ where correlated with the partial pressure measured by the reference optode (*p*_*O*2_) at the upper outlet of the lysimeter (Figure 1). We used the test series for oxygen because of its large measurement range. For the different concentration plateaus (see Figure 2) the mean values of *a*_1_ and the partial pressure are plotted (Figure 3) including the 3-fold standard deviation for both *a*_1_ and *p*_*O*2_.

Table 1 presents the fit results of *a*_1_ = (*c*_1_ ± *δc*_1_) *p*_O2_ + (*c*_2_ ± *δc*_2_) for all monitoring planes (see Figure 2) where c*_i_*are fit parameters with standard errors *δc_i_*, *R²* is correlation coefficient.

Both Figure 3 and Table 1 confirm our linearity assumption over the whole measurement range. Furthermore, the coefficients *c*_2_ are negative. Due to purging the tube sensors by air the regression crosses zero if the oxygen concentration inside the lysimeter exceeds the one in the purging gas.

**Figure 4.**
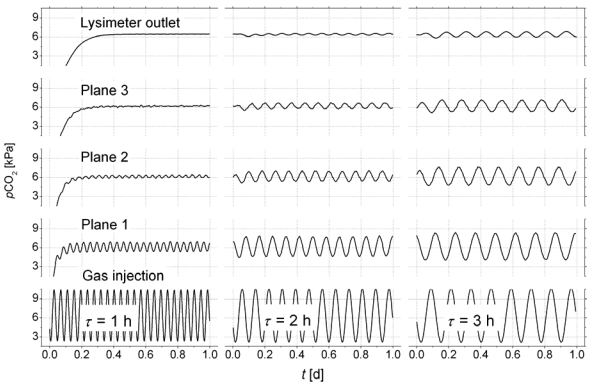
Convective-dispersive propagation of oscillating pCO_2_ - input concentrations (mixtures with dry air) inside the lysimeter.

To demonstrate the ability of the new technique to capture fluctuating concentrations in a multi-component gas as typical for natural systems we mix CO_2_ and dry air dynamically to obtain an oscillating partial pressure: *P*_*CO*2_(*t*)|_*x*=0_ = *A* + *B* sin(2*π t/τ*) (*A* = 6 kPa, *B* = 4 kPa, *τ* [h] – oscillation period). The flow rate of the gas mixture was adjusted to 0.5 L/min. Figure 4 shows the dampening of the signal along the flow path, which demonstrates the accuracy of the measurement.

The first test was performed with an oscillation period of *τ* = 1h, which was completely smoozed inside the sediment. Using an oscillation period of two hours, the reference sensor on the top of the lysimeter started to see the oscillation. For *τ* = 3h the signal on top was sufficient to analyse cross-correlations of the signals between the monitoring planes. The lag of the first correlation maximum marks the mean travel time of the concentration wave for the distance between two monitoring planes i, j: Δ*t_1,2_* = 27.3 min, Δ*t_2,3_* = 28.6 min. Using the distances between the monitoring planes (see Figure 1) the mean distance velocity can be calculated (*v_1,2_* = 0.71 cm/min, *v_2,3_* = 0.70 cm/min).

The porosity of φ = 0.35 was calculated from bulk density of the sediment. Together with the known cross sections of the lysimeter the volumetric gas flow was calculated based on the velocities *v_1,2_*, and *v_2,3_*. We obtained *Q_1,2_* = 0.578 L/min, and *Q_2,3_* = 0.579 L/min, which differ by less than 1 %.

The comparison with the actually applied flow rate of 0.5 L/min indicates an overestimation of the flow. However, due to the complex geometry of the pore space, it can be assumed that not the entire air-filled cross section contributes to flow. Based on our measurements we could estimate an ‘effective’ porosity of 0.31.

## 5. Conclusions

This study presents a novel *in situ* sensor concept. The membrane-based sensors have demonstrated its long term stability in a lab-lysimeter over a couple of years. Using such sensors with tubular geometry it is possible to measure an average gas concentration value over a certain line with negligible impact on the environment by the sensor. As demonstrated by monitoring of O_2_ and CO_2_ in a lysimeter, this technique is highly attractive for monitoring the gas dynamics in soil. To expand the number of measurable gases (e.g., CH_4_, H_2_S) further experimental work is necessary.

If such sensors are installed in a specific pattern (e.g., regular, hierarchic, site specific), it is possible to calculate a meaningful (representative) average of gas concentrations over a larger area. Therefore, the measuring tube can replace a large number of individual sensors, reducing the cost for representative measurements by previous methods. It will also be possible to gain scale-depended insights into the spatial variability of gas behavior (formation, migration) in saturated and unsaturated porous media. Another advantage of the sensor is the possibility to use ordinary and easily available tube materials after a simple calibration.

Potential technical applications for membrane-based gas sensors are environmental remediation (e.g., measuring of O_2_- and CO_2_-distributions in the heterogeneous subsurface for aerobic biodegradation) like biosparging or bioventing of organic contaminants in ground- and seepage water and landfill monitoring.

Due to the fast answer of such line-sensor networks the technology could be also advantageous for safety monitoring of CO_2_-sequestration, gas pipelines or sewers. The principle can be applied in pure liquids for monitoring of e.g., surface waters and boreholes.

## Figures and Tables

**Table 1.** Fit data for the regression lines *a*_1_ = (*c*_1_ ± *δc*_1_) *p*_O2_ + (*c*_2_ ± *δc*_2_) in Figure 3 (*c_i_* – fit parameters with standard errors *δc_i_, R²* – correlation coefficient).

**Monitoring plane**	***c*_1_[*Pa s*^-1^/*kPa*]**	***δc*_1_[*Pa s*^-1^/*kPa*]**	***c*_2_[*Pa s*^-1^]**	***δc*_2_[*Pa s*^-1^]**	***R^2^***
Plane 3	0.7293	0.0091	-15.23	0.46	0.999
Plane 2	0.7743	0.0102	-16.52	0.53	0.999
Plane 1	0.7447	0.0095	-15.68	0.49	0.999
